# Survival and Its Correlates in Multiple Sclerosis Patients under a Universal Health Insurance Program in Taiwan: An 18-Year Nationwide Cohort Study

**DOI:** 10.3390/healthcare11111551

**Published:** 2023-05-25

**Authors:** Chun-Ming Liao, Chia-Yu Chen, Pei-Tseng Kung, Wei-Yin Kuo, Hui-Chuan Chuang, Wen-Chen Tsai

**Affiliations:** 1 Graduate Institute of Public Health, China Medical University, Taichung 406040, Taiwan; jm.liao@msa.hinet.net (C.-M.L.); u103050003@cmu.edu.tw (C.-Y.C.); 2 Genetic and Rare Disease Center, China Medical University Hospital, China Medical University, Taichung 404327, Taiwan; a92592@mail.cmu.edu.tw; 3 Department of Healthcare Administration, Asia University, Taichung 41354, Taiwan; 4 Department of Medical Research, China Medical University Hospital, China Medical University, Taichung 404327, Taiwan; 5 Department of Health Services Administration, College of Public Health, China Medical University, Taichung 406040, Taiwan; u100050853@cmu.edu.tw; 6 Center for General Education, China Medical University, Taichung 406040, Taiwan

**Keywords:** multiple sclerosis, survival, cause of death, cohort study, Taiwan

## Abstract

Despite the global decline in the standardized mortality rate of multiple sclerosis (MS), recent research on MS patient survival, especially in Taiwan, remains limited. This study aimed to investigate survival, mortality causes, and associated factors among MS patients in Taiwan. The Taiwan National Health Insurance Research Database was used as the primary data source, and a Cox proportional hazard model was employed to estimate and analyze factors related to survival. We analyzed data from 1444 MS patients diagnosed between 2000 and 2018. Age at diagnosis was positively correlated with the risk of death. Among the 190 patients who died, the leading causes of disease-related deaths were nervous system diseases (n = 83, 43.68%), followed by respiratory system diseases and certain infectious and parasitic diseases. The 8-, 13-, and 18-year survival rates for MS patients were 0.97, 0.91, and 0.81, respectively. This study highlights that the MS patient’s socioeconomic status, environmental factors, comorbidity severity, and related medical variables were not significantly associated with survival.

## 1. Introduction

Multiple sclerosis (MS) is a prevalent disease worldwide [1,2]. MS is not considered a hereditary disease; however, many high-risk genetic variations have been identified [3]. Studies have shown that environmental factors, particularly Epstein–Barr virus infection and smoking, contribute to an increased incidence of MS, while vitamin D supplementation has been associated with a decreased risk [4]. However, the supplementation of vitamin D has been considered a challenging task with limitations and the need for medical supervision [5]. Additionally, research has demonstrated the beneficial effects of estrogens and progestins in experimental autoimmune encephalomyelitis (EAE), such as neural self-repair and reduced disease incidence [6]. These findings suggest that the pathogenesis of MS involves complex interactions between genetic susceptibility and environmental triggers, including hormonal, genetic, and environmental influences, as well as gene–environment interactions and epigenetic mechanisms [7].

Although the pathogenesis of MS is not clear, this disease exhibits specificity with respect to age, gender, and temporal trends. MS occurs most frequently between the ages of 20 and 50 and affects women at approximately three times the rate of men [2,8]. Among children, the ratio of girls to boys with MS is more than two-fold, and a study on pediatric multiple sclerosis (ped-MS) reports that the increasing trend of female adult-onset MS is also evident in girls. Furthermore, a significant proportion of pediatric MS patients develop marked cognitive impairment and sustained physical disability at a greater rate than adult-onset MS patients [9]. The gender ratio differs greatly among age groups, and unlike the two-fold difference between girls and boys, MS affects men and women almost equally among middle-aged and elderly patients, especially those over 50 years old [10].

A longitudinal study from Norway, spanning 60 years, showed that the life expectancy of MS patients is approximately 7 years shorter than that of the general population, and the mortality rate has increased three-fold [11]. Similar conclusions have been drawn in numerous other studies worldwide [12,13,14]. Beta interferon treatment was associated with a lower mortality risk among people with relapsing-onset MS [15]. There are various types of new disease-modifying drugs for MS and their indications. These medications aim to improve the quality of life and significantly extend patients’ lifespans by reducing episodes of central nervous system demyelination and inflammation [16]. Some studies have investigated the effectiveness of Chinese herbal medicines; however, they do not discuss the risk of death after treatment [17]. In fact, the analysis of the risk of death in MS patients receiving alternative therapies is rarely mentioned in the literature.

Few studies have explored the risk of death and related factors in Taiwanese MS patients. A previous study examined the impact of disease-modifying therapies (DMTs) on the risk of death in Taiwanese MS patients, but the data only extended up to 2008 and require updating [18]. Three MS disease reports based on Taiwan’s national data after 2000 as a sample do not mention any analysis of the risk of death in MS patients [19,20,21]. An MS epidemiology report in Taiwan showed a slight decrease in the fatality rate of MS from 2001 to 2015 [22]. The Global Burden of Disease (GBD) meta-analysis also revealed that Taiwan’s standardized mortality rate for MS decreased by 23.9% from 1990 to 2016, which was greater than the global decline [1]. Hence, there is a need to investigate the risk of death in Taiwanese MS patients.

Taiwan has had a National Health Insurance (NHI) system since 1 March 1995 [23]. As of 2015, 95.8% of the medical institutions in Taiwan, comprising 27,728 NHI-designated medical institutions, were participating in the NHI program, and the coverage rate of the population was 99.68% [24,25]. The government health authorities track and provide care for MS patients, according to the “Prevention of Rare Diseases and Orphan Drug Act” [26]. Several studies have shown that Taiwan has an adequate and universally accessible healthcare system. Taiwan’s NHI fully covers the medical expenses of patients with MS and the cost of medicines [27,28]. The NHI system enhances the accessibility of Taiwan’s medical resources, enabling the National Health Insurance Research Database (NHIRD) data provided by the Ministry of Health and Welfare of Taiwan to reflect the true status of the source population better. These data are based on research and are representative of the entire population of Taiwan.

This study aimed to investigate the risk of death and its correlates in MS patients in Taiwan using the nationwide NHIRD data. We anticipate that our findings will not only provide a reference for the clinical care of MS in Taiwan but also improve the reliability of the global MS disease literature.

## 2. Materials and Methods

### 2.1. Data Sources

In this study, NHIRD and the Cause of Death data provided by the Ministry of Health and Welfare of Taiwan from 2000 to 2018 were utilized as the data source for the study population. Additionally, the Household Register File, established and provided by the Ministry of the Interior of Taiwan, was linked to the study data. All study subjects were followed up until 31 December 2018, to ensure complete follow-up data.

### 2.2. Subjects

MS patients in Taiwan were diagnosed by physicians who utilized the latest McDonald criteria available for that year [29] and subsequently registered in NHIRD. Physicians then applied to Taiwan’s Health Promotion Administration, Ministry of Health and Welfare to identify the MS patients [30]. After identification, physicians then applied to the National Health Insurance (NHI) to identify records in the “Registry of Patients with Catastrophic Illnesses” [25]. This two-stage process of identification and registration was helpful for confirming the patient’s illness and diagnosis of MS. Registration of MS disease in the “Registry of Patients with Catastrophic Illnesses” has a 5-year validity period [31], which prevents the progression and inspection of patient diseases. Diagnostic differences can be attributed to advances in equipment and technology. Therefore, we ensured the reliability and effectiveness of the conditions for our observation objects. We included only those MS patients who were continuously diagnosed with MS within the validity period (every 5 years) and excluded those who became undiagnosed.

The present study enrolled subjects with confirmed MS diagnoses (ICD-9-CM code: 340, ICD-10-CM code: G35) between 1 January 2001 and 31 December 2015. To ensure accurate identification, we traced the patient’s first main diagnosis code for outpatient visits, emergency department visits, and hospitalizations in the “details of ambulatory care orders”. This approach helped to confirm the diagnosis and eliminate gaps between the time of completing the “Registry of Patients with Catastrophic Illnesses” and the patient’s first diagnosis. To prevent confounding factors, we used the year 2000 as the wash-out period. Moreover, we excluded patients aged ≤ 14 years, as their disease course may differ from that of adults [32].

Patient identification in the NHIRD, household registration, and cause of death files was encrypted to ensure privacy. The study was conducted in compliance with the 1964 Declaration of Helsinki and its amendments, and informed consent was waived by the Research Ethics Committee. This study was approved by the China Medical University & Hospital Research Ethics Center (IRB No. CMUH108-REC2-084). No identifying information was disclosed to ensure patient privacy.

### 2.3. Variable Description and Definition

The survival of subjects was the primary outcome and was tracked until 31 December 2018. Death (all-cause death) was recorded based on the death time registered in the “cause of death” data. A subject was considered an event if they died before the end of 2018, and if they survived, they were censored. Fourteen independent variables were tested: The annual newly diagnosed patients (>14 years) were classified into three time periods, (2001–2005, 2006–2010, and 2011–2015). Demographic characteristics included sex (female or male), age at diagnosis (15–24, 25–34, 35–44, 45–54, 55–64, and ≥65 years), and marital status (unmarried, married, and other). Socioeconomic status was determined by education level (elementary school and under, junior high school, senior high/vocational school, and junior college/university and above) and monthly salary (≤TWD 22,800, TWD 22,801–45,800, and ≥TWD 45,801). Environmental factors were measured by the degree of urbanization of the patient’s residential area, which was categorized into seven levels according to Liu et al. [33]. The subject’s health condition, including the severity of comorbidity, was based on the Charlson comorbidity index (CCI) modified by Deyo et al., where the ICD-9-CM primary and secondary diagnostic codes of the subject’s medical treatment were converted into numerical weighted scores [34]. The subjects were divided into 0 points, 1 point, 2 points, and ≥3 points according to the severity of comorbidities. The presence or absence of other catastrophic illnesses was also recorded (yes or no). Related medical variables included the patient’s outpatient, emergency, and hospitalization visits within three years after a new diagnosis. We observed whether the patient received disease-modifying therapies (DMTs) that they took three or more times in any year during the observation period within three years after the patient was newly diagnosed (yes or no). Chinese medicinal treatment was defined as the cumulative use of Chinese medicine services (Chinese medicine and acupuncture, etc.) for more than 30 days (times) each year during the observation period within three years after the patient was newly diagnosed (yes or no). Physiotherapy treatment was recorded as positive if the patient underwent more than 30 sessions in any year during the observation period within three years after the patient was newly diagnosed (yes or no). Ownership of hospital (public and private) and hospital level (medical center, regional hospital, and district hospital) were the main characteristics of the treatment hospitals.

### 2.4. Analytical Methods

This retrospective cohort study utilized SAS 9.4 (SAS Institute Inc., Cary, NC, USA) for statistical analysis. The aim of this research was to examine the survival of the subjects, which served as the dependent variable. Initially, the chi-squared test was performed to investigate the characteristics of subjects in various 5-year periods (2001–2005, 2006–2010, 2011–2015) and to verify the associations of the independent variables, which included demographic characteristics, socioeconomic status, environmental factors, health condition, related medical variables, and hospital characteristics. The study explored whether there were differences in the number of subjects diagnosed in each 5-year period (*p* < 0.05).

The causes of death, including the number of deaths from various diseases and non-disease deaths, were analyzed and expressed as percentages. The log-rank test was used to analyze the survival of the subjects across the three 5-year periods (2001–2005, 2006–2010, and 2011–2015) and to determine the association between independent variables and control variables with survival. The number of subjects, their percentages, and the survival probability distribution for each variable exhibited significant differences (*p* < 0.05).

To further examine the survival status and its correlates in the subjects, the Cox proportional hazard model (Cox PH regression) was used. First, the unadjusted relative risk of each variable was estimated without controlling for any variables, and the unadjusted hazard ratio (HR) was calculated. Then, the relative risk of each variable was analyzed with the control of other variables (adjusted HR), and related factors were analyzed (*p* < 0.05).

The survival model was used to analyze the survival curves of all subjects diagnosed from 2001 to 2015, and the three diagnosis intervals (2001–2005, 2006–2010, and 2011–2015) were examined separately to draw three curves. In instances where missing values occurred randomly, the complete case method was employed [35,36]. Overall, these analytical methods allowed us to conduct a comprehensive investigation of the survival and associated factors of MS patients in Taiwan.

## 3. Results

The study compared subject characteristics and the use of immunomodulatory interferon drugs across different time periods. Chi-squared tests were conducted to verify the characteristics of subjects in the three 5-year periods (2001–2005, 2006–2010, 2011–2015). Significant differences were found in the distribution of the number of subjects among 6 variables (*p* < 0.05), including survival status, marital status, socioeconomic status, and health condition (Table 1). In 2015, nine types of interferons were approved for MS in Taiwan [37], but only six drugs met our screening criteria. While the tables provide a general overview of disease-modifying therapies (DMTs), the specific mention of interferons in the text does not exclude other DMTs. Our study acknowledges a broader range of DMTs available for MS treatment, using interferons as an example rather than an exhaustive list. Table 2 shows the use of immunomodulatory interferon drugs by subjects in 2005, 2010, and 2015, with “Interferon beta-1a” being the most commonly used drug. The drug “Interferon beta-1a” was taken at 145 frequency (69.05%), 199 frequency (60.12%), and 221 frequency (64.81%), respectively. The next most common was “Interferon beta-1b” (24.76%, 27.19%, 11.14%) and “Fingolimod” (1.51%, 16.42%).

**Figure 1:**
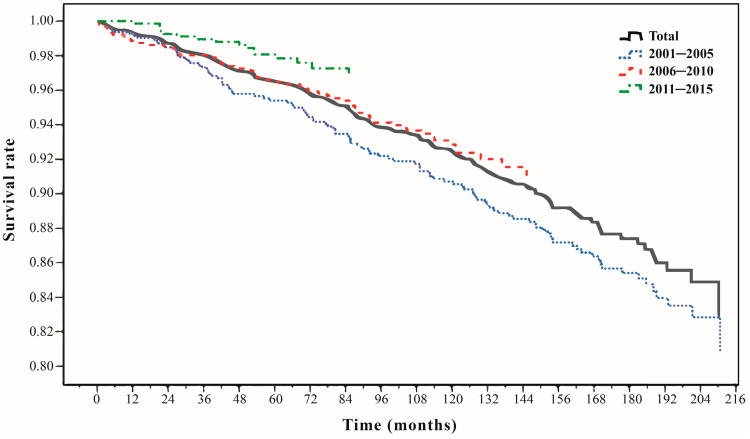
The survival curves of subjects in 2001–2018 and in the three periods.

The causes of death of the subjects were analyzed, including the number of deaths and their percentages (%), and survival curves were plotted. Table 3 shows that out of a total of 190 deaths during the observation period (2001–2018), 6 were non-disease deaths, and the remaining 184 deaths were from various diseases. Multiple disease categories were identified, with nervous system diseases, including MS, accounting for the majority of deaths (n = 83, 43.68% of all deaths). Survival curves of subjects from 2001 to 2018 were drawn with the survival model, and the survival rate was 0.83. The annual newly diagnosed patients (>14 years) were categorized into three time periods, and the 8-year (96-month), 13-year (156-month), and 18-year (216-month) survival rates were analyzed (Figure 1).

To explore the survival status and its correlates in the subjects, the risk of death was compared using the log-rank test. Significant contributions to the risk of death were found for 11 variables, including the time periods, subject’s demographic characteristics, socioeconomic status, environmental factors, health condition, physical therapy, and the characteristics of the main treatment hospital (*p* < 0.05) (Table 4). All subjects were tracked until 31 December 2018.

After controlling for relevant variables, we investigated the mortality risk of subjects using the Cox proportional hazard model (Cox PH regression), and the results are presented in Table 4. The analysis revealed that the time periods, age at diagnosis, presence of other catastrophic illnesses, and hospital level had a significant impact on the risk of death (HR) (*p* < 0.05), and the other 10 variables did not show statistically significant HRs. Specifically, the risk of death increased with age at diagnosis, with the 35–44-year-old group having 2.52 times the risk of death of the 15–24-year-old group (95% CI = 1.15–5.51), and the ≥65-year-old group having the highest risk, at 14.21 (95% CI = 5.56–35.85). The group with other catastrophic illnesses had a 2.14 times (95% CI = 1.57–2.93) higher risk of death than the group without other catastrophic illnesses. The district hospital group had a 2.31 times (95% CI = 1.36–3.91) higher risk of death than the medical center group. Moreover, the 2011–2015 diagnosis group had a 57% lower risk of death than the 2001–2005 group (HR = 0.43, 95% CI = 0.24–0.76). However, the use of DMTs (HR = 1.17, 95% CI = 0.85–1.60), traditional Chinese medicines (HR = 0.85, 95% CI = 0.60–1.18), physical therapy (HR = 1.16, 95% CI = 0.68–2.00), and the other three medical variables were not statistically significant.

## 4. Discussion

This empirical database study reveals that the risk of death for MS patients in Taiwan increases with age at diagnosis. The highest risk of death was observed in the ≥65-year-old group, with a hazard ratio (HR) of 14.21, followed by three age groups in the range 35–64 years old (HRs of 3.47, 5.11, and 11.85). MS patients with other catastrophic illnesses also had a significantly higher risk of death than those without such illnesses (HR = 2.14). On the other hand, patient demographic characteristics, socioeconomic status, environmental factors, comorbidity severity, and related medical variables did not show statistically significant HRs. Our findings are consistent with the results of an epidemiological report in Taiwan, which revealed a sharp increase in mortality with age over a 15-year period [22]. Similarly, studies conducted in other countries have shown that age is a significant predictor of mortality in MS patients [12,14]. We also learned from previous research reports that during the 15-year observation period, MS patients had an overall mortality rate of 1334 people per 100,000 person-years. In comparison, the standardized population mortality rate in 2015 was 431.5 people per 100,000 population in Taiwan, indicating a mortality ratio of approximately three times higher in MS patients [22]. A previous study in Taiwan identified monthly salary and interferon drug treatment as significant factors affecting mortality [18]. However, the observed discrepancy in our study regarding the association between risk factors (e.g., older age, rural residence, and lower economic status) and other MS-related mortality may be attributed to variations in sampling time and the number of control variables.

We used NHIRD as our data source, which is maintained by the Taiwanese government and is the most complete research data available in Taiwan. Our study is one of the hundreds of studies published in peer-reviewed international journals that have used NHIRD data since 2012 [38,39]. To control for confounding factors affecting the probability of patient death, we used the NHIRD’s data structure to connect multiple databases and included 14 control variables in our multivariate statistical analysis. These variables included patient demographic characteristics, socioeconomic status, environmental factors, health condition, related medical variables, characteristics of the main treatment hospitals, and time periods. The use of multiple variables has been recommended in previous studies [28]. We also used a five-year-period control variable to eliminate the effects of long-term MS diagnosis and treatment progress gaps. While physiotherapy treatment showed significant differences in survival according to the log-rank test and unadjusted Cox regression analysis, this effect did not persist after controlling for other variables in our multivariate analysis. The variables in Table 4 (HR) did not show statistically significant results. This may suggest that the effect of physiotherapy on MS patient mortality is partially excluded after controlling for related variables. Our study’s use of 14 variables to control for confounding factors is rare in many long-term survival analyses and provides a high degree of credibility to our results.

In our study, the most common cause of death was MS (n = 83, 43.68% of all deaths), which was slightly lower than the results of follow-up studies in Norway and Denmark for over ten years (50%, 55.4%) [40,41]. However, pneumonia was the second most common cause of death (5.79%), slightly higher than the rate in the aforementioned study (5.1%). Other studies have also shown that in addition to MS and pneumonia, sepsis and malignant neoplasms are the next most common causes of death [14,42], which is consistent with our findings. These results suggest that, in clinical treatment, DMTs mainly suppress the patient’s autoimmune attack response. Although most DMTs are interferon drugs, they can lead to adverse reactions and a decline in immune system function [43].

Epidemiological reports of MS in Taiwan and worldwide have indicated a decline in standard incidence and fatality rates in recent years [1,22]. Firstly, we observed that the district hospital group had a higher risk of death than the medical center group. One possible reason for this disparity could be the insufficient nursing resources in district hospitals compared to medical centers [44]. We also observed a 57% lower risk of death (HR = 0.43) in the 2011–2015 group than in the 2001–2005 group in terms of patient diagnosis, and the difference was statistically significant. This may be due to advancements in the diagnosis and treatment of MS. However, the HR value we obtained was not entirely reliable because the observation time for the 2011–2015 group was relatively short (maximum of 8 years), coinciding with the survival time of most patients in this period. As a result, the survival period is likely longer than the observation time, leading to right-censored data bias in survival analysis [45] and weight calculation methods [46] or extending the observation time to correct for this deviation.

Interferon drug treatment can significantly reduce the mortality rate of MS patients [15,18]. We believe that among older MS patients in Taiwan or those suffering from other catastrophic illnesses, as well as many other groups at high risk of death, some may choose to receive interferon drug treatment and traditional Chinese medicine, resulting in a bias in the selection of independent variables. Therefore, it is impossible to use the Cox regression analytical method to control for the related variables and clarify their association with the death of the dependent variables [47]. Given the limitations of our study, further analysis is not possible. We recommend that future studies use the paired study method of an observation group and a control group for comparative analysis [47].

The current study provides valuable insights into the causes and risk factors of mortality among MS patients in Taiwan. However, there are still some limitations that need to be addressed in future research. One limitation is the lack of data on certain important factors such as patients’ lifestyles, comorbidities, and treatment adherence. Future studies should consider collecting such data to provide a more comprehensive understanding of the risk factors associated with mortality among MS patients in Taiwan. Secondly, this study only observed the Taiwanese population with MS and did not involve other countries or ethnic groups. Future research could further compare the mortality rate and survival time of MS patients among different countries or ethnic groups to determine the potential impact of cultural, social, and environmental differences on the prognosis of MS patients. Additionally, using the Cox proportional hazard model in our study is subject to limitations associated with baseline data availability and assumptions of proportional hazards. Future studies should consider collecting comprehensive data on these factors and exploring alternative statistical approaches that can account for time-varying covariates. Finally, future research could explore the impact of different types of treatments on mortality rates among MS patients, including newer therapies that have been developed in recent years. Overall, the current study provides a foundation for future research to build upon and enhance our understanding of mortality risk factors among MS patients in Taiwan.

## 5. Conclusions

Although the standard incidence rate and fatality rate of MS in Taiwan have declined slightly in recent years, our empirical study found that patient demographic characteristics (sex and marital status), socioeconomic status (education level and monthly salary), environmental factors, comorbidity severity, and related medical variables (DMTs, Chinese medicine treatment, and physiotherapy treatment) were not significantly associated with survival. Therefore, we propose that healthcare institutions in Taiwan embrace a comprehensive approach to meet the overall care needs of MS patients.

## Figures and Tables

**Table 1:** The characteristics of subjects in the three periods.

	Total		2001~2005 ^a^		2006~2010 ^a^		2011~2015 ^a^		*p* Value ^b^
	N	%	N	%	N	%	N	%	
All subjects	1444	100.00	488	33.80	508	35.18	448	31.02	
Survival status ^c^									<0.001
censor	1254	86.84	372	76.23	450	88.58	432	96.43	
death	190	13.16	116	23.77	58	11.42	16	3.57	
Sex		0.00		0.00		0.00		0.00	0.845
Female	1113	77.08	372	76.23	395	77.76	346	77.23	
Male	331	22.92	116	23.77	113	22.24	102	22.77	
Age at diagnosis ^d^									0.263
15–24	298	20.64	87	17.83	104	20.47	107	23.88	
25–34	424	29.36	142	29.10	140	27.56	142	31.70	
35–44	323	22.37	116	23.77	115	22.64	92	20.54	
45–54	236	16.34	86	17.62	91	17.91	59	13.17	
55–64	115	7.96	38	7.79	40	7.87	37	8.26	
≥65	48	3.32	19	3.89	18	3.54	11	2.46	
Marital status ^d^									0.001
Unmarried	633	43.84	191	39.14	210	41.42	232	51.67	
Married	691	47.85	260	53.28	249	49.11	182	40.53	
Other	117	8.10	36	7.38	48	9.47	33	7.35	
Unknown	3	0.21	1	0.20	0	0.00	2	0.45	
Education level ^d^									<0.001
Elementary school and under	185	12.81	100	20.41	55	11.14	30	6.52	
Junior high school	256	17.73	91	18.57	90	18.22	75	16.30	
Senior high/vocational school	604	41.83	202	41.22	220	44.53	182	39.57	
Junior college/university and above	363	25.14	85	17.35	120	24.29	158	34.35	
Unknown	36	2.49	12	2.45	9	1.82	15	3.26	
Monthly salary (TWD) ^d,e^									0.014
≤22,800	843	58.38	304	62.30	303	59.65	236	52.68	
22,801–45,800	439	30.40	143	29.30	143	28.15	153	34.15	
≥45,801	162	11.22	41	8.40	62	12.20	59	13.17	
Urbanization level of residence area ^d^									0.932
Level 1	455	31.51	156	32.16	154	30.26	145	32.23	
Level 2	503	34.83	158	32.58	195	38.30	150	33.34	
Level 3	219	15.17	74	15.26	76	14.93	69	15.33	
Level 4	170	11.77	60	12.37	55	10.81	55	12.22	
Level 5	14	0.97	5	1.03	4	0.79	5	1.11	
Level 6	41	2.84	15	3.09	13	2.55	13	2.89	
Level 7	38	2.63	16	3.30	11	2.16	11	2.44	
Unknown	4	0.28	1	0.21	1	0.20	2	0.44	
CCI ^d,f^									<0.001
0	920	63.71	287	58.81	329	64.76	304	67.86	
1	265	18.35	80	16.39	99	19.49	86	19.20	
≥2	259	17.94	121	24.80	80	15.75	58	12.95	
Other catastrophic illnesses ^d,g^									<0.001
No	1192	82.55	368	75.41	430	84.65	394	87.95	
Yes	252	17.45	120	24.59	78	15.35	54	12.05	
DMTs ^h^									0.858
No	659	45.64	293	60.04	197	38.78	169	37.72	
Yes	785	54.36	195	39.96	311	61.22	279	62.28	
Chinese medicine treatment									0.927
No	989	68.49	337	69.06	345	67.91	307	68.53	
Yes	455	31.51	151	30.94	163	32.09	141	31.47	
Physiotherapy treatment									0.363
No	1379	95.50	470	96.31	480	94.49	429	95.76	
Yes	65	4.50	18	3.69	28	5.51	19	4.24	
Ownership of hospital ^d^									0.027
Public	855	59.21	311	63.99	294	57.76	250	55.67	
Private	585	40.51	174	35.80	214	42.04	197	43.88	
Unknown	4	0.28	1	0.21	1	0.20	2	0.45	
Hospital level ^d^									0.328
Medical center	1023	70.84	344	70.78	373	73.28	306	68.15	
Regional hospital	351	24.31	114	23.46	116	22.79	121	26.95	
District hospital	66	4.57	27	5.55	19	3.73	20	4.45	
Unknown	4	0.28	1	0.21	1	0.20	2	0.45	

^a^ 2001~2005, 2006~2010, 2011~2015: The annual newly diagnosed patients (>14 years) were broken up into three time periods. ^b^ *p* value: The chi-squared test was used to examine the characteristics of subjects in various 5-year periods (2001~2005, 2006~2010, and 2011~2015) to explore whether there were differences in the number of subjects diagnosed (*p* < 0.05). ^c^ Survival status: The occurrence of death (all-cause death) was based on the death time registered in the “cause of death” data. If the subject died before the end of 2018, it was an event, and if the subject survived, it was a censor. ^d^ Age at diagnosis, marital status, education level, monthly salary, urbanization level of residence area, CCI, other catastrophic illnesses, ownership of hospital, and hospital level: The status of the patients when they were newly diagnosed. ^e^ TWD: New Taiwan dollar. ^f^ CCI: Charlson comorbidity index. ^g^ Other catastrophic illnesses: catastrophic illnesses excluding MS. ^h^ DMTs: Disease-modifying therapies for multiple sclerosis patients.

**Table 2:** The number of users of immunomodulatory interferon drugs by subjects in the three years.

Pharmaceutical Ingredients	ATC ^a^	2005		2010		2015	
		N	%	N	%	N	%
Total frequency ^b^		210	100.00	331	100.00	341	100.00
Interferon beta-1a	L03AB07	145	69.05	199	60.12	221	64.81
Fingolimod	L04AA27	0	0	5	1.51	56	16.42
Interferon beta-1b	L03AB08	52	24.76	90	27.19	38	11.14
Glatiramer acetate	L03AX13	13	6.19	35	10.57	25	7.33
Natalizumab	L04AA23	0	0	0	0	1	0.29
Mitoxantrone	L01DB07	0	0	2	0.60	0	0

^a^ ATC: Anatomical therapeutic chemical classification system. ^b^ Total frequency: Number of times subjects used medicine in one year.

**Table 3:** The causes of death of the subjects.

Causes of Death/Disease Category/Disease	People	%
Total of people	190	100.00%
Non-disease deaths	6	3.16%
Accidental deaths	3	1.58%
Suicides	3	1.58%
Disease deaths	184	96.84%
Diseases of the nervous system		
Multiple sclerosis	83	43.68%
Intracranial and intraspinal phlebitis and thrombophlebitis	4	2.11%
Diseases of the respiratory system		
Pneumonia, unspecified organism	11	5.79%
Certain infectious and parasitic diseases		
Sepsis, unspecified	6	3.16%
Neoplasms		
Malignant neoplasm of unspecified site of unspecified female breast	4	2.11%
Malignant neoplasm of bronchus or lung, unspecified, unspecified side	2	1.05%
Symptoms, signs and abnormal clinical and laboratory findings, not elsewhere classified		
Respiratory arrest	3	1.58%
Other specified symptoms and signs involving the circulatory and respiratory systems	2	1.05%
Diseases of the circulatory system		
Atherosclerotic heart disease of native coronary artery without angina pectoris	3	1.58%
Diseases of the genitourinary system		
Urinary tract infection, site not specified	3	1.58%
Diseases of the musculoskeletal system and connective tissue		
Systemic lupus erythematosus, organ or system involvement unspecified	3	1.58%
Endocrine, nutritional and metabolic diseases		
Type 2 diabetes mellitus with other diabetic neurological complication	3	1.58%
Other (57 disease)	57	29.99%

**Table 4:** Risk of Death and Survival Analysis in Patients with MS (End of 2018).

	Bivariate Analysis							Survival Analysis					
	Total		Censor ^a^		Death ^a^			Unadjusted		Adjusted			
	N	%	N	%	N	%	*p* Value ^b^	HR	*p*-Value	HR	95% CI		*p*-Value ^c^
All subjects	1444	100.00	1254	86.84	190	15.15							
Time periods ^d^							<0.001						
2001~2005 (ref)	488	33.80	372	76.23	116	23.77		1.00	-	1.00	-	-	-
2006~2010	508	35.18	450	88.58	58	11.42		0.67	0.018	0.75	0.53	1.07	0.113
2011~2015	448	31.02	432	96.43	16	3.57		0.37	<0.001	0.43	0.24	0.76	0.004
Sex							0.585						
Female (ref)	1113	77.08	956	76.20	144	75.80		1.00	-	1.00	-	-	-
Male	331	22.92	298	23.80	46	24.20		1.10	0.585	1.36	0.95	1.94	0.089
Age at diagnosis ^ e^							<0.001						
15~24 (ref)	298	20.64	287	96.31	11	3.69		1.00	-	1.00	-	-	-
25~34	424	29.36	398	93.87	26	6.13		1.64	0.169	1.39	0.65	2.97	0.396
35~44	323	22.37	281	87.00	42	13.00		3.47	<0.001	2.52	1.15	5.51	0.021
45~54	236	16.34	192	81.36	44	18.64		5.11	<0.001	3.31	1.49	7.34	0.003
55~64	115	7.96	74	64.35	41	35.65		11.85	<0.001	7.26	3.18	16.55	<0.001
≥65	48	3.32	22	45.83	26	54.17		21.06	<0.001	14.21	5.63	35.85	<0.001
Marital status ^e^							<0.001						
Unmarried (ref)	633	43.84	599	94.63	34	5.37		1.00	-	1.00	-	-	-
Married	691	47.85	560	81.04	131	18.96		3.43	<0.001	1.15	0.70	1.87	0.587
Other	117	8.10	93	79.49	24	20.51		3.89	<0.001	0.98	0.51	1.87	0.940
Unknown	3	0.21	3	100.00	0	0.00							
Education level ^e^							<0.001						
Elementary school and under (ref)	185	12.81	122	65.95	63	34.05		1.00	-	1.00	-	-	-
Junior high school	256	17.73	210	82.03	46	17.97		0.54	0.002	1.24	0.79	1.94	0.347
Senior high/vocational school	604	41.83	547	90.56	57	9.44		0.27	<0.001	0.71	0.45	1.11	0.133
Junior college/university and above	363	25.14	341	93.94	22	6.06		0.19	<0.001	0.73	0.40	1.33	0.305
Unknown	36	2.49	32	88.89	4	11.11							
Monthly salary (TWD) ^e,f^							0.004						
≤22,800 (ref)	843	58.38	709	84.10	134	15.90		1.00	-	1.00	-	-	-
22,801~45,800	439	30.40	394	89.75	45	10.25		0.65	0.011	0.76	0.52	1.09	0.131
≥45,801	162	11.22	151	93.21	11	6.79		0.47	0.015	0.54	0.28	1.04	0.064
Urbanization level of residence area ^e^							0.022						
Level 1 (ref)	455	31.51	408	89.67	47	10.33		1.00	-	1.00	-	-	-
Level 2	503	34.83	432	85.88	71	14.12		1.43	0.056	1.40	0.96	2.06	0.083
Level 3	219	15.17	195	89.04	24	10.96		1.04	0.889	1.03	0.62	1.73	0.904
Level 4	170	11.77	138	81.18	32	18.82		1.94	0.004	1.36	0.84	2.21	0.213
Level 5	14	0.97	10	71.43	4	28.57		3.35	0.020	1.16	0.36	3.74	0.804
Level 6	41	2.84	37	90.24	4	9.76		0.95	0.922	0.66	0.23	1.91	0.442
Level 7	38	2.63	32	84.21	6	15.79		1.49	0.355	1.08	0.44	2.66	0.873
Unknown	4	0.28	4	100.00	0	0.00							
CCI ^e,g^							<0.001						
0 (ref)	920	63.71	837	90.98	83	9.02		1.00	-	1.00	-	-	-
1	265	18.35	227	85.66	38	14.34		1.70	0.007	1.04	0.68	1.57	0.869
≥2	259	17.94	190	73.36	69	26.64		3.00	<0.001	1.16	0.80	1.68	0.441
Other catastrophic illnesses ^e,h^							<0.001						
No (ref)	1192	82.55	1083	90.86	109	9.14		1.00	-	1.00	-	-	-
Yes	252	17.45	171	67.86	81	32.14		3.39	<0.001	2.14	1.57	2.93	<.0001
DMTs ^i^							0.112						
No (ref)	659	45.64	555	84.22	104	15.78		1.00	-	1.00	-	-	-
Yes	785	54.36	699	89.04	86	10.96		0.79	0.114	1.17	0.85	1.60	0.335
Chinese medicine treatment							0.278						
No (ref)	989	68.49	853	86.25	136	13.75		1.00	-	1.00	-	-	-
Yes	455	31.51	401	88.13	54	11.87		0.84	0.279	0.85	0.60	1.18	0.327
Physiotherapy treatment							0.003						
No (ref)	1379	95.50	1205	87.38	174	12.62		1.00	-	1.00	-	-	-
Yes	65	4.50	49	75.38	16	24.62		2.13	0.004	1.16	0.68	2.00	0.581
Ownership of hospital ^e^							0.042						
Public (ref)	855	59.21	729	85.26	126	14.74		1.00	-	1.00	-	-	-
Private	585	40.51	524	89.57	61	10.43	0.730	0.043	0.910	0.65	1.26	0.556	0.730
Unknown	4	0.28	4	100.00	0	0.00							
Hospital level ^e^							<0.001						
Medical center (ref)	1023	70.84	906	88.56	117	11.44		1.00	-	1.00	-	-	-
Regional hospital	351	24.31	299	85.19	52	14.81		1.38	0.052	1.09	0.76	1.58	0.634
District hospital	66	4.57	48	72.73	18	27.27		2.66	<0.001	2.31	1.36	3.91	0.002
Unknown	4	0.28	4	100.00	0	0.00							

^a^ Censor or Death: The occurrence of death (all-cause death) was based on the death time registered in the “cause of death” data. If the subject died before the end of 2018, it was an event, and if the subject survived, it was a censor. ^b^ *p* value: The log-rank test was used to analyze whether the subjects survived, and there was a significant difference in the number of subjects for each variable (*p* < 0.05). ^c^ *p* value: The Cox proportional hazard model was used to estimate the unadjusted/adjusted relative risk of each variable with controlling for any variables and to calculate the HR (*p* < 0.05). ^d^ 2001~2005, 2006~2010, 2011~2015: The annual newly diagnosed patients (>14 years) were broken up into three time periods. ^e^ Age at diagnosis, marital status, education level, monthly salary, urbanization level of residence area, CCI, other catastrophic illnesses, ownership of hospital, and hospital level: The status of the patients when they were newly diagnosed. ^f^ TWD: New Taiwan dollar. ^g^ CCI: Charlson comorbidity index. ^h^ Other catastrophic illnesses: catastrophic illnesses excluding MS. ^i^ DMTs: Disease-modifying therapies for multiple sclerosis patients.

## Data Availability

Regarding the data availability, data were obtained from the National Health Insurance Research Database published by the Ministry of Health and Welfare, Taiwan. Due to legal restrictions imposed by the Taiwan government relating to the Personal Information Protection Act, the database cannot be made publicly available. All researchers can apply to use the databases to conduct their studies. Requests for data can be sent as a formal proposal to the Health and Welfare Data Science Center of the Ministry of Health and Welfare, Taiwan (http://www.mohw.gov.tw/EN/Ministry/Index.aspx (accessed on 1 July 2022)). No raw data are allowed to be removed from the Health and Welfare Data Science Center. The restrictions prohibited the authors from making the minimal dataset publicly available.

## References

[1] Wallin M.T., Culpepper W.J., Nichols E., Bhutta Z.A., Gebrehiwot T.T., Hay S.I., Khalil I.A., Krohn K.J., Liang X., Naghavi M. (2019). Global, regional, and national burden of multiple sclerosis 1990–2016: A systematic analysis for the global burden of disease study 2016. Lancet Neurol..

[2] National Institute of Neurological Disorders and Stroke (2019). Multiple Sclerosis Information Page. https://www.ninds.nih.gov/Disorders/All-Disorders/Multiple-Sclerosis-Information-Page#disorders-r1.2022/1/142022.

[3] Dyment D.A., Ebers G.C., Sadovnick A.D. (1981). Genetics of multiple sclerosis. Lancet Neurol..

[4] Pugliatti M., Harbo H.F., Holmøy T., Kampman M.T., Myhr K.M., Riise T., Wolfson C. (2008). Environmental risk factors in multiple sclerosis. Acta Neurol. Scand..

[5] Feige J., Moser T., Bieler L., Schwenker K., Hauer L., Sellner J. (2020). Vitamin d supplementation in multiple sclerosis: A critical analysis of potentials and threats. Nutrients.

[6] El-Etr M., Ghoumari A., Sitruk-Ware R., Schumacher M. (2011). Hormonal influences in multiple sclerosis: New therapeutic benefits for steroids. Maturitas.

[7] Voskuhl R.R., Gold S.M. (2012). Sex-related factors in multiple sclerosis: Genetic, hormonal and environmental contributions. Nat. Rev. Neurol..

[8] McAlpine D., Compston A. (2005). Mcalpine’s Multiple Sclerosis.

[9] Ghezzi A., Banwell B., Boyko A., Amato M.P., Anlar B., Blinkenberg M., Boon M., Filippi M., Jozwiak S., Ketelslegers I. (2010). Meeting review: The management of multiple sclerosis in children: A european view. Mult. Scler. J..

[10] Miller D.H., Leary S.M. (2007). Primary-progressive multiple sclerosis. Lancet Neurol..

[11] Lunde H.M.B., Assmus J., Myhr K.-M., Bø L., Grytten N. (2017). Survival and cause of death in multiple sclerosis: A 60-year longitudinal population study. J. Neurol. Neurosurg. Psychiatry.

[12] Kingwell E., van der Kop M., Zhao Y., Shirani A., Zhu F., Oger J., Tremlett H. (2011). Relative mortality and survival in multiple sclerosis: Findings from british columbia, canada. J. Neurol. Neurosurg. Psychiatry.

[13] Brønnum-Hansen H., Koch-Henriksen N., Stenager E. (2004). Trends in survival and cause of death in danish patients with multiple sclerosis. Brain.

[14] Phadke J.G. (1987). Survival pattern and cause of death in patients with multiple sclerosis: Results from an epidemiological survey in north east scotland. J. Neurol. Neurosurg. Psychiatry.

[15] Kingwell E., Leray E., Zhu F., Petkau J., Edan G., Oger J., Tremlett H. (2019). Multiple sclerosis: Effect of beta interferon treatment on survival. Brain.

[16] Barten L.J., Allington D.R., Procacci K.A., Rivey M.P. (2010). New approaches in the management of multiple sclerosis. Drug Des. Dev. Ther..

[17] Song L., Zhou Q.-H., Wang H.-L., Liao F.-J., Hua L., Zhang H.-F., Huang L.-B., Lin Y., Zheng G.-Q. (2017). Chinese herbal medicine adjunct therapy in patients with acute relapse of multiple sclerosis: A systematic review and meta-analysis. Compl. Med..

[18] Tsai C.-P., Lee C.T.-C. (2013). Impact of disease-modifying therapies on the survival of patients with multiple sclerosis in taiwan, 1997–2008. Clin. Drug Investig..

[19] Lai C.-H., Tseng H.-F. (2009). Population-based epidemiological study of neurological diseases in taiwan: I. Creutzfeldt-jakob disease and multiple sclerosis. Neuroepidemiology.

[20] Fang C.-W., Wang H.-P., Chen H.-M., Lin J.-W., Lin W.-S. (2020). Epidemiology and comorbidities of adult multiple sclerosis and neuromyelitis optica in taiwan, 2001–2015. Mult. Scler. Relat. Disord..

[21] Hsu C.-Y., Ro L.-S., Chen L.-J., Chang C.-W., Chang K.-H., Wu I.-H., Lin A., Hsiao F.-Y. (2021). Epidemiology, treatment patterns and healthcare utilizations in multiple sclerosis in taiwan. Sci. Rep..

[22] Liao C.-M., Kuo W.-Y., Kung P.-T., Chuan C.-H., Tsai W.-C. (2022). Epidemiological investigation of multiple sclerosis and related medical utilisation in taiwan. Mult. Scler. J..

[23] Health Promotion Administration (2015). National Health Insurance Act. https://law.moj.gov.tw/ENG/LawClass/LawAll.aspx?pcode=L0060001.

[24] Ministry of Health and Welfare (2016). The National Health Insurance Statistics, 2016. https://www.nhi.gov.tw/english/Content_List.aspx?n=17BC32CF4F3F289E&topn=616B97F8DF2C3614.

[25] Huang S.-K. (2015). 2015–2016 National Health Insurance Annual Report.

[26] Health Promotion Administration (2015). Prevention of Rare Diseases and Orphan Drug Act. https://www.hpa.gov.tw/EngPages/Detail.aspx?nodeid=1058&pid=6031.

[27] Sharma A., Jacob A., Tandon M., Kumar D. (2010). Orphan drug: Development trends and strategies. J. Pharm. Bioallied Sci..

[28] Hsu J.C., Wu H.-C., Feng W.-C., Chou C.-H., Lai E.C.-C., Lu C.Y. (2018). Disease and economic burden for rare diseases in taiwan: A longitudinal study using taiwan’s national health insurance research database. PLoS ONE.

[29] World Health Organization (2008). Atlas: Multiple Sclerosis Resources in the World 2008.

[30] Lin J.-D., Lin L.-P., Hung W.-J. (2013). Reported numbers of patients with rare diseases based on ten-year longitudinal national disability registries in taiwan. Res. Dev. Disabil..

[31] National Health Insurance Administration (2019). Scope of Catastrophically Ill of National Health Insurance. https://www.nhi.gov.tw/Resource/webdata/1059_2_10000406%E9%87%8D%E5%A4%A7%E5%82%B7%E7%97%85%E7%AF%84%E5%9C%8D%E8%A1%A8-%E7%BD%AE%E7%B6%B2%E7%AB%99.pdf.

[32] Duignan S., Brownlee W., Wassmer E., Hemingway C., Lim M., Ciccarelli O., Hacohen Y. (2019). Paediatric multiple sclerosis: A new era in diagnosis and treatment. Dev. Med. Child Neurol..

[33] Liu C.-Y., Hung Y.-T., Chuang Y.-L., Chen Y.-J., Weng W.-S., Liu J.-S., Liang K. (2006). Incorporating development stratification of taiwan townships into sampling design of large scale health interview survey. J. Health Manag..

[34] Deyo R.A., Cherkin D.C., Ciol M.A. (1992). Adapting a clinical comorbidity index for use with icd-9-cm administrative databases. J. Clin. Epidemiol..

[35] Shrive F.M., Stuart H., Quan H., Ghali W.A. (2006). Dealing with missing data in a multi-question depression scale: A comparison of imputation methods. BMC Med. Res. Methodol..

[36] Nakai M., Chen D.-G., Nishimura K., Miyamoto Y. (2014). Comparative study of four methods in missing value imputations under missing completely at random mechanism. Open J. Stat..

[37] Food and Drug Administration (2017). 2017 Rare Disease Prevention and Treatment and Medication Act Drug Annual Report. https://www.hpa.gov.tw/EngPages/EngTopicList.aspx?nodeid=1072.

[38] Hsieh C.-Y., Su C.-C., Shao S.-C., Sung S.-F., Lin S.-J., Yang Y.-H.K., Lai E.C.-C. (2019). Taiwan’s national health insurance research database: Past and future. Clin. Epidemiol..

[39] Lin L.-Y., Warren-Gash C., Smeeth L., Chen P.-C. (2018). Data resource profile: The national health insurance research database (nhird). Epidemiol. Health.

[40] Smestad C., Sandvik L., Celius E. (2009). Excess mortality and cause of death in a cohort of norwegian multiple sclerosis patients. Mult. Scler. J..

[41] Koch-Henriksen N., Brønnum-Hansen H., Stenager E. (1998). Underlying cause of death in danish patients with multiple sclerosis: Results from the danish multiple sclerosis registry. J. Neurol. Neurosurg. Psychiatry.

[42] Hirst C., Swingler R., Compston D., Ben-Shlomo Y., Robertson N.P. (2008). Survival and cause of death in multiple sclerosis: A prospective population-based study. J. Neurol. Neurosurg. Psychiatry.

[43] Koriem K.M.M. (2017). Corrigendum to ‘multiple sclerosis: New insights and trends’. Asian Pac. J. Trop. Biomed..

[44] Liang Y.-W., Chen W.-Y., Lee J.-L., Huang L.-C. (2012). Nurse staffing, direct nursing care hours and patient mortality in taiwan: The longitudinal analysis of hospital nurse staffing and patient outcome study. BMC Health Serv. Res..

[45] Kronek L.-P., Reddy A. (2008). Logical analysis of survival data: Prognostic survival models by detecting high-degree interactions in right-censored data. Bioinformatics.

[46] Gerds T.A., Schumacher M. (2006). Consistent estimation of the expected brier score in general survival models with right-censored event times. Biom. J..

[47] Rosenbaum P.R., Rubin D.B. (1983). The central role of the propensity score in observational studies for causal effects. Biometrika.

